# Simultaneous ORIF for bilateral comminuted proximal humerus fractures: Case report in an elderly patient

**DOI:** 10.1016/j.ijscr.2019.10.061

**Published:** 2019-11-01

**Authors:** Joseph Maalouly, Dany Aouad, Nabil Dib, Antonios Tawk, Georges El Rassi

**Affiliations:** Department of Orthopedic Surgery and Traumatology Saint Georges University Medical Center, Balamand University, P.O. Box 166378, St Georges Street, Achrafieh, Beirut, 1100 2807, Lebanon

**Keywords:** Case report, Shoulder, Trauma, ORIF

## Abstract

**Introduction:**

Bilateral proximal humerus fractures are rather rare and occur due to high energetic traumas in young patients and even low energetic traumas in older patients having osteoporosis. Open reduction and internal fixation (ORIF) is the most popular treatment for this type of fracture while other types of treatment such as arthroplasty or conservative treatment are also, but less commonly used.

**Presentation of case:**

This report presents the case of an 80 years old female patient with bilateral proximal humerus fractures and a history of hypothyroidism. The patient was treated with simultaneous open reduction and internal fixation and presented a normal and full-range motion in both shoulders after follow-up.

**Discussion:**

Frequency of proximal humerus fractures has increased with the increase in life expectancy. Osteoporotic people are at higher risk. Around five percent of all extremity fractures are humerus proximal end fractures for people below 40 years old and 76 % for cases of people over 40 years old, with females being more susceptible. ORIF is the treatment of choice for such fractures. Replacement arthroplasty, minimally invasive techniques and conservative management are other options depending on fracture characteristics.

**Conclusion:**

This is an interesting rare case of simultaneous bilateral proximal humerus fractures in an elderly female patient, treated operatively with satisfactory results on follow up.

## Highlights

• Rare case of traumatic bilateral proximal humerus fractures.
• Simultaneous bilateral open reduction and internal fixation showed good recovery.
• Post op care is of great importance for patient recovery and return to daily activities.

## 1 Introduction

Around five to eight percent of fracture cases include proximal humerus fractures [1]. In young patients, this type of fracture is often caused due to high energetic traumas, as well as in subjects presenting with convulsions and electrocution. However, in osteoporotic cases, even low energy traumas such as falling down can result in humerus proximal fractures [2]. Bilateral proximal humerus fractures are rather rare. They can often occur as results of epileptic episodes, electrocution or extreme traumas [3]. In most cases, proximal humerus fractures are surgically treated [1]. We present a case of an elderly woman who sustained trauma post fall to both her shoulders, found to have simultaneous bilateral comminuted proximal humeral fractures. Patient underwent ORIF, with satisfactory results, returning to normal daily activities progressively on follow up. This type of presentation is rarely reported, especially due to trauma. Surgical management is usually the adapted modality of treatment, which in this case was done for both shoulders simultaneously instead of two staged surgeries, with a satisfactory outcome. To note, the work has been reported in line with the SCARE criteria [4].

## 2 Case report

**Fig. 1:**
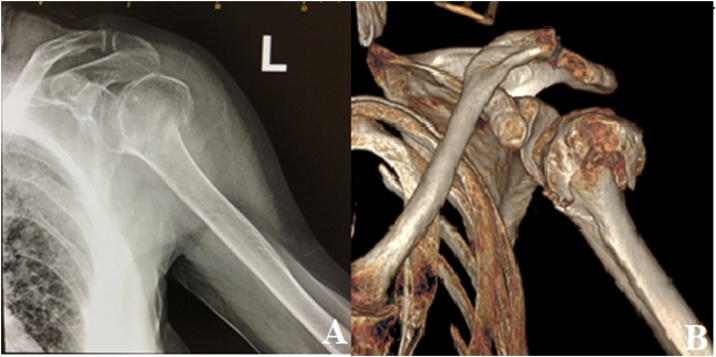
(A) X-Ray radiograph of the left shoulder showing a comminuted proximal humerus fracture. (B) CT 3D reconstruction of the left shoulder showing a Neer III displaced fracture of the humeral neck.

**Fig. 2:**
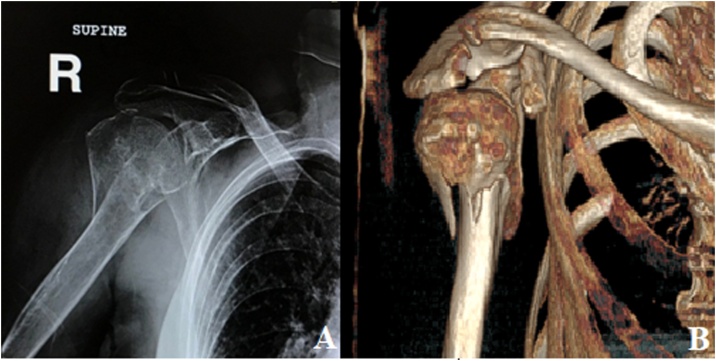
(A) X-Ray radiograph of the right shoulder showing a comminuted proximal humerus fracture (B) CT scan with 3D reconstruction of the Right shoulder showing a Neer IV fracture of the surgical neck and proximal shaft of the humerus involving the greater tubercle. The head has rotated laterally and cranially.

This is the case of a 80 year old female patient with a history of hypothyroidism on adequate treatment who presented one hour after a fall from height. She reports falling with the elbows flexed and a direct impact to the face and shoulders. Patient reports living alone, able to perform all activities of daily living prior to trauma. On physical examination, patient was found to have decreased and painful bilateral shoulder ROM, with intact proximal and distal neurovascular structures. Patient suffered from bilateral proximal humerus fractures seen on plain radiographs and on CT scan with 3D reconstruction (Fig. 1, Fig. 2).

**Fig. 3:**
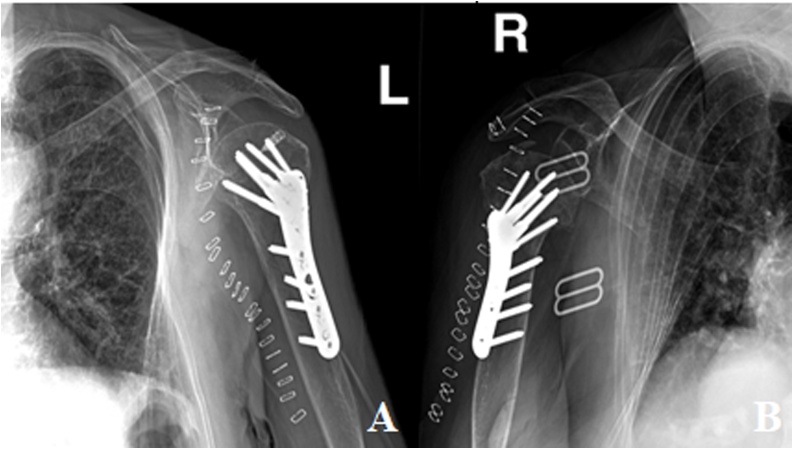
(A) Post-operative X-ray radiograph of the left shoulder showing good alignement post-ORIF of the proximal humerus fracture using plate and ten screws (B) Post-operative X-ray radiograph of the right shoulder showing good alignement post-ORIF of the proximal humerus fracture using plate and ten screws.

The fractures were treated with open reduction and internal fixation. Using a deltopectoral approach, beach chair position and arm rest, dissection and identification of cephalic vein, further dissection done reaching the bone. Biceps was identified then tenotomy was done. Reduction under fluoroscopy after impaction of the fracture in a valgus position. The bones were reduced and fixed with K-wires for provisional fixation, using abduction and traction, and then a proximal anatomical plate was placed anterolaterally, lateral to the bicipital groove to neutralize any medial comminution under fluoroscopic guidance with repair of the medial and lateral tuberosities. The plate was fixed with 10 screws (Fig. 3). To note, the supraspinatus tendon was found ruptured intra-operatively and was repaired using an anchor on the left side.

At four months follow up, after multiple physiotherapy sessions, the patient had full range of motion restored and was back to her daily routine activities, without any symptoms.

## 3 Discussion

The frequency of proximal humerus fractures has increased with the increase in the average life expectancy. Osteoporotic people are at higher risk of such fractures. Around five percent of all extremity fractures are humerus proximal end fractures for cases of people below 40 years old and 76 % for cases of people over 40 years old. In addition, females are three times more at risk than males [2]. Open reduction and internal fixation (ORIF) is the most popular treatment for this type of fracture. However, other types of treatment are also, but less commonly used. These treatments include replacement arthroplasty, minimally invasive techniques and non-operative treatment [3]. Jaiswal et al. (2013) performed ORIF on a 40 years old male patient with bilateral proximal humerus fractures using proximal humerus locked plates. The outcomes were satisfactory and the patient was able to return to her daily activities [3]. Kara et al. (2013) reported on the cases of two women (62 and 72 years old) with the same case. An operation was performed on both women after giving them Tramadol to reduce the fracture. Both cases occurred due to low energetic traumas because of bone density decrease due to osteoporosis [2]. Ellanti & Harrington (2012) reported on a 56 years old female who underwent ORIF (with threaded pins and tension band suture) as treatment for her right shoulder and Hemiarthroplasty for her left shoulder. After two years, both shoulders showed similar satisfactory results. However, the patient herself expressed a preference for the right shoulder stating it felt more secure and natural [5]. Rodrigues-Crolay (2016) employed conservative treatment with two hanging casts and a radiological treatment conducted during the first week and after two additional weeks. Satisfactory results were reported, showing that conservative treatment can also be a viable treatment option in such cases [1]. A study by Dixit et al. (2013) examined the Nationwide Inpatient Sample (NIS) in the United States in order to make a comparison between ORIF and arthroplasty treatments. Of the 1413 patients between the years 1998 and 2013, 662 underwent ORIF treatment while only 68 underwent arthroplasty. The study found that patients who underwent ORIF were younger but that no significant difference in terms of complication rates existed between the two procedures [6]. Generally, ORIF is desired in young patients for bone preservation. However, ORIF has been associated in other studies with higher reoperation rates despite showing better functional outcomes [7]. Spross et al. (2018) developed an algorithm that can be employed to determine the type of treatment used based on the patient’s age, type of fracture and bone quality. The treatments used were ORIF, RTSA and hemiarthroplasty (HA). The results showed that the treatments were successful and ensured a normal life quality for patients after being treated. According to the algorithm, ORIF was employed for young and active patients as well as older patients with good bone quality. However, ORIF was not a viable option in some cases of young patients because of not being able to reduce the fracture in a stable manner. In these cases, HA was employed but did not offer promising results. RTSA was employed for elderly patients. Conservative treatment was considered for young and elderly patients who has -part fracture [8]. According to Dinopolous [9], the presumed mechanism of action in his patient was fall on outstretched hands with abduction due to the reflex to protect her face. Often, one hand reaches the ground earlier than the other and thus bears the brunt of the trauma; but in that case both hands reached simultaneously [10]. Bilateral proximal humerus fractures are either due to forceful muscular contraction, as in electrocutions or seizures, or deceleration forces from the trauma [9]. It has been suggested that resistance training as well as learning how to fall may decrease fracture occurrence [11]. In our case, a reverse shoulder prosthesis may have been an option. However, it is associated with an increase in the morbidity rate and increase in the time of surgery; thus more risk exists for bleeding, infection and other morbidities.

## 4 Conclusion

Proximal humerus fractures are common with ongoing debate as to what the best treatment modality is, depending on the patient age. Bilateral proximal humerus fracture are very rare, with direct trauma to both shoulders simultaneously as a mechanism of action. In our case of an elderly patient, bilateral proximal humerus fracture was treated by simultaneous open reduction using mini-invasive technique and internal fixation with anchor on the left side for rotator cuff repair. This surgery is associated with less morbidities and less risk to the patient with excellent results. In such cases, it is important to prepare hemiarthroplasty prosthesis and reverse shoulder prosthesis as backup in case of failure of open reduction and internal fixation due to irreducibility of fracture of lack of good bone stock.

## Sources of funding

No funds were received in support of this study.

## Ethical approval

Ethics committee has given approval for publication.

## Consent

Written informed consent was obtained from the patient for publication of this case report and accompanying images. A copy of the written consent is available for review by the Editor-in-Chief of this journal on request.

No identity identifiers are present whatsoever in the manuscript.

## Author’s contribution

Joseph Maalouly: contributed to the writing and editing of this article.

Dany Aouad: contributed to the writing of this article and the submission.

Nabil Dib: contributed to the writing and editing of the article along with the figures.

Antonios Tawk: contributed to the editing and finishing of the article.

Georges El Rassi: contributed with the case, surgical management and editing of the article.

## Registration of research studies

This case has been registered in the IRB committee of St Georges Hospital University Hospital.

## Guarantor

Dr Dany Aouad.

## Provenance and peer review

Not commissioned, externally peer-reviewed.

## Declaration of Competing Interest

The authors declare no conflict of interest regarding the publication of this article.
